# A novel CsbZIP26–CsSEP4–CsSPL18 regulatory module governs gynostemium morphology and floral architecture in *Cymbidium sinense*

**DOI:** 10.1093/hr/uhaf329

**Published:** 2025-12-08

**Authors:** Zengyu Lin, Chuqiao Lu, Yibing Wang, Yonglu Wei, Jie Gao, Jie Li, Qi Xie, Jianpeng Jin, Yanmei Sun, Wei Zhu, Genfa Zhu, Fengxi Yang

**Affiliations:** Guangdong Key Laboratory of Ornamental Plant Germplasm Innovation and Utilization, Environmental Horticulture Research Institute, Guangdong Academy of Agricultural Sciences, Guangzhou 510640, China; State Key Laboratory of Tropical Crop Breeding, Shenzhen Branch, Guangdong Laboratory of Lingnan Modern Agriculture, Key Laboratory of Synthetic Biology, Ministry of Agriculture and Rural Affairs, Agricultural Genomics Institute at Shenzhen, Chinese Academy of Agricultural Sciences, Shenzhen 518120, China; Guangdong Key Laboratory of Ornamental Plant Germplasm Innovation and Utilization, Environmental Horticulture Research Institute, Guangdong Academy of Agricultural Sciences, Guangzhou 510640, China; Guangdong Key Laboratory of Ornamental Plant Germplasm Innovation and Utilization, Environmental Horticulture Research Institute, Guangdong Academy of Agricultural Sciences, Guangzhou 510640, China; Guangdong Key Laboratory of Ornamental Plant Germplasm Innovation and Utilization, Environmental Horticulture Research Institute, Guangdong Academy of Agricultural Sciences, Guangzhou 510640, China; Guangdong Key Laboratory of Ornamental Plant Germplasm Innovation and Utilization, Environmental Horticulture Research Institute, Guangdong Academy of Agricultural Sciences, Guangzhou 510640, China; Guangdong Key Laboratory of Ornamental Plant Germplasm Innovation and Utilization, Environmental Horticulture Research Institute, Guangdong Academy of Agricultural Sciences, Guangzhou 510640, China; Guangdong Key Laboratory of Ornamental Plant Germplasm Innovation and Utilization, Environmental Horticulture Research Institute, Guangdong Academy of Agricultural Sciences, Guangzhou 510640, China; Guangdong Key Laboratory of Ornamental Plant Germplasm Innovation and Utilization, Environmental Horticulture Research Institute, Guangdong Academy of Agricultural Sciences, Guangzhou 510640, China; Guangdong Key Laboratory of Ornamental Plant Germplasm Innovation and Utilization, Environmental Horticulture Research Institute, Guangdong Academy of Agricultural Sciences, Guangzhou 510640, China; Guangdong Key Laboratory of Ornamental Plant Germplasm Innovation and Utilization, Environmental Horticulture Research Institute, Guangdong Academy of Agricultural Sciences, Guangzhou 510640, China; Guangdong Key Laboratory of Ornamental Plant Germplasm Innovation and Utilization, Environmental Horticulture Research Institute, Guangdong Academy of Agricultural Sciences, Guangzhou 510640, China; Guangdong Key Laboratory of Ornamental Plant Germplasm Innovation and Utilization, Environmental Horticulture Research Institute, Guangdong Academy of Agricultural Sciences, Guangzhou 510640, China

## Abstract

Floral organ formation plays an essential role in *Cymbidium sinense* reproductive development and serves as a key determinant of their ornamental traits. During the domestication and natural evolution of *C. sinense*, numerous floral organ variant cultivars have emerged, among which many floral morphological variations arise from abnormal development of the gynostemium, a reproductive organ. These gynostemium variant (GV) cultivars not only exhibit enhanced commercial appeal but also provide a unique model for investigating floral morphogenesis and evolutionary diversification. In this study, we identified single nucleotide polymorphisms (SNPs) in the promoter region of *CsSEP4* closely linked to GV through genome-wide association studies. Functional analyses of *CsSEP4* revealed that it played a crucial role in the development of gynostemium. Yeast one-hybrid (Y1H) and dual-luciferase reporter assays indicated that the CsbZIP26 transcription factor binds to the *CsSEP4* promoter and activates its expression in normal flowers, whereas the SNP mutations from ACGTG to ATGTG or ACGTA of the *CsSEP4* promoter were detected in GV lines, which resulted in the inability of CsbZIP26 to bind and regulate the expression of *CsSEP4*. Furthermore, DNA affinity purification sequencing (DAP-seq) and Y1H experiments identified *CsSPL18* as a direct downstream target of CsSEP4. Genetic evidence also demonstrated that CsSEP4 orchestrates gynostemium development by positively activating *CsSPL18* expression. Collectively, our results revealed that the *CsbZIP26–CsSEP4–CsSPL18* regulatory module governs the development of stamen gynostemium to regulate flower morphology in *C. sinense*. These findings provide insight into the molecular mechanisms underlying gynostemium development in orchids and establish a molecular framework for further elucidating orchid diversity and evolution.

## Introduction

Throughout the processes of plant domestication and natural evolution, distinctive developmental characteristics have progressively evolved, such as multiple-lip and multiple-gynoecium floral structures. These adaptive traits enable plants to undergo natural selection and environmental adaptation, which have captivated evolutionary biologists [1]. In model plants like *Arabidopsis thaliana* and *Antirrhinum majus*, the flower pattern has been proposed by the ‘ABCDE model’ [2–4]. This framework has been pivotal in clarifying the molecular mechanisms governing floral morphogenesis and its evolutionary diversification. However, in contrast to the model plants, the unique reproductive biology of orchids has given rise to distinctive evolutionary innovations, such as the expansion and contraction of gene families, and specialized developmental trajectories of reproductive organs [5, 6]. In Orchidaceae, the ‘HOT model’, the ‘perianth (P) code model’, and the ‘Orchid code’ have been widely recognized as the hypotheses to explain labellum specification and perianth differentiation [7–9]. Notably, orchid gene duplication events have facilitated regulatory divergence, enabling novel protein complexes that orchestrate orchid-specific floral ontogeny [10, 11]. For instance, expansions in B-class (*AP3*) and E-class gene lineages are linked to the evolutionary development of the specialized labellum and gynostemium (column) in orchids [12, 13], while *CeSEP2* is highly correlated with the peloric flower and can cause special labellum in *Cymbidium ensifolium* [14]. Additionally, SEPALLATA (SEP) proteins likely interact with other MADS-box factors to regulate the development of the ovule [15]. Despite extensive findings, the regulatory mechanisms of orchid-specific floral organ structure and development are still far from fully understood. Especially, the gynostemium (column), a fused organ of the androecium and gynoecium, is a specialized structure that characterizes the morphology of flower organs in Orchidaceae.

The advancement of genome-wide association studies (GWAS) technology has not only deepened our comprehension of the evolution of key agronomic traits but also pinpointed critical genetic loci for crop genetic enhancement. Through GWAS analysis, several key gene loci are found in *Phalaenopsis* and *Cymbidium*, including functional conserved genes and species-specific gene loci with novel functions, which have provided new clues for the genetic regulation of key agronomic traits in Orchidaceae plants [16]. During floral development in plants, the cooperative regulation of multiple genes is involved [17]. This process is typically mediated by transcription factors that activate target gene expression by binding to specific DNA sequences, thereby collectively contributing to floral morphogenesis. Well established as conserved transcription factors, basic leucine zipper (*bZIP*) genes form an extensive family in plants that orchestrate diverse biological processes, notably floral development [18–20]. However, compared with *Arabidopsis*, their role in orchid floral specification remains unclear. In *Arabidopsis*, reports have shown that *bZIPs* genes, *TGA1* to *TGA7*, *PERIANTHIA* (*PAN*), *TGA9*, and *TGA10*, participate in flower pattern formation and anther development [21–24]. In tobacco, the knockdown of *TGA2.1* leads to stamen-to-petal transformations [25]. In rice, *OsTGA10* governs tapetal development and male fertility [24]. Intriguingly, reports have shown that in *Phalaenopsis*, a *TGA1a*-like bZIP TF may be a crucial factor in the formation of peloric mutant flowers [26]. Hence, *bZIP* genes are postulated to exert conservative effects on floral organ development during floral morphogenesis.

Squamosa promoter-binding protein-like (*SPL*) genes, as plant-specific transcription factors, are involved in various developmental processes and exert significant functions, such as the male fertility in reproductive organs [27]. In *Arabidopsis*, *AtSPL8* maintains male fertility and gynoecium development, while *AtSPL7* is essential for anther and pollen fertility [28–30]. MADS-box gene *AtSOC1* promotes flowering by regulating the *AtSPL3*, *AtSPL4*, and *AtSPL5* genes through direct binding to their promoters [31, 32]. Furthermore, in *Cucumis sativus*, *CsSPL* acts as an adaptor to orchestrate anther and ovule development [33, 34], and in rice, five genes (*OsSPL6/8/13/14/16*) have been identified as regulators of inflorescence architecture and panicle apical abortion [35]. Similar genes have been implicated in orchid flower development. For example, previous reports regarding the *SPL* gene families in Orchidaceae imply that they are comprehensively involved in flower development [36, 37]. Additionally, *SPL* genes may regulate the development of multitepal organs in *Cymbidium goeringii* [38]. However, the functional characterization of orchid *SPL* genes is limited, with no direct evidence linking specific *SPL* members to floral organ identity determination or developmental regulatory networks.

Floral morphology and novel floral structures are critical for studying the evolution of phenotypic traits in orchids and meeting market demand. In this study, through genetic analyses, we demonstrated that the *CsSEP4* gene is essential for gynostemium determinacy in *Cymbidium sinense*. To further elucidate the regulatory mechanisms of *CsSEP4* in floral structure development, we identified single nucleotide polymorphisms (SNPs) in the promoter region of the *CsSEP4* gene through GWAS. Using yeast one-hybrid (Y1H) assays, we screened out CsbZIP26, an upstream regulatory factor of *CsSEP4*. Subsequent analyses revealed that SNP variations in the *CsSEP4* promoter between wild-type (WT) and gynostemium variant (GV) varieties alter CsbZIP26-mediated transcriptional activation efficiency, thereby affecting the role of *CsSEP4* in maintaining gynostemium development. Furthermore, DAP-seq and RNA-seq analyses determined *CsSPL18* as a direct downstream target gene of CsSEP4. Further analyses revealed that CsSEP4 functions in gynostemium determinacy by direct binding to the promoter of *CsSPL18*, thereby enhancing its expression. Collectively, our study proposes that the *CsbZIP26–CsSEP4–CsSPL18* regulatory module orchestrates floral patterning of gynostemium in *C. sinense*. This mechanistic model reveals that the specification of floral organ morphologies is part of a hierarchical gene regulatory network, providing an evolutionary-developmental foundation to elucidate the origins of morphological specialization and diversification in orchid floral architecture.

## Results

### The SNPs in the promoter of *CsSEP4* are identified in *C. sinense*

**Figure 1:**
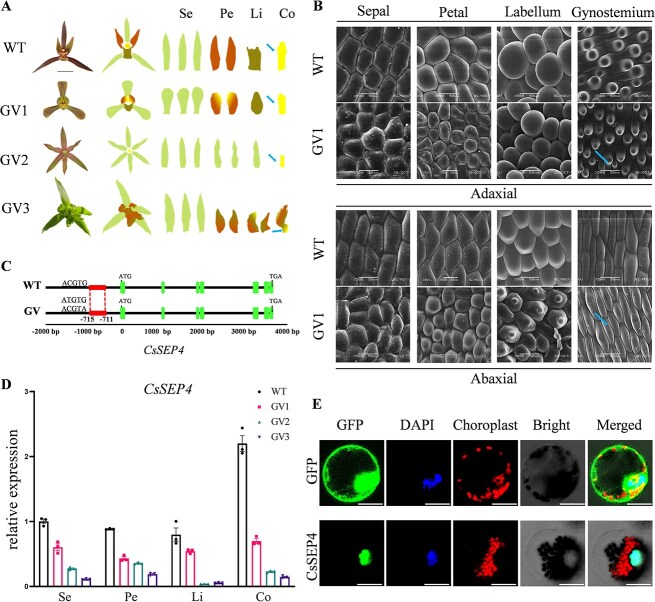
Expression pattern of *CsSEP4* and observation of floral cell shape in *C. sinense* varieties between WT (normal floral morphology) and GV. (A) WT represents normal flower morphology, and GV1\2\3 represent three principal phenotypes of GVs of *C. sinense*. Bar: 1 cm. (B) Cell shapes of flowers of WT and GV1 were observed by cryo-SEM. Adaxial and abaxial epidermis in sepals, petals, labellum, and gynostemium. Arrows indicate changes in epidermal cells. Bars: 50 μm. (C) The SNPs between −711 and −715 in the promoter of *CsSEP4* in WT and GV, and the CsSEP4 coding sequence (CDS), are depicted. (D) RT-qPCR analyzed the expression of *CsSEP4* in WT and GV. Se indicates sepal; Pe, petal; Li, labellum; Co, gynostemium (Column). Data represent the mean ± SEM of three biological replicates, significance is evaluated by the two-way analysis of variance, and *P* values are indicated. (E) Subcellular locations of CsSEP4 in protoplasts of *C. sinense*. GFP represents gene localization; chloroplast represents chloroplast self-luminescence. Scale bars: 20 μm.

GV, a common floral morphological variation type in Chinese *Cymbidium*, is characterized by developmental abnormalities of the gynostemium and mainly divided into three types (Fig. 1A). The first type exhibits a constricted gynostemium enveloped by outer tepals, with anther-like structure developing at the apex of the tepals (GV1); the second type involves severe gynostemium reduction, showing a simple flat structure in the center of the flower lacking both pollinia and anther cap (GV2); the third type shows absence of the gynostemium in the floral medial region, accompanied by fused pollen-like structures at the tepal apex (GV3). Correspondingly, the cell structure also undergoes changes in the variant types. Epidermal cells developed from flattened to cylindrical morphologies from sepals to gynostemium in WT. In contrast, in GV1, petal and labellum cells exhibited cylindrical morphologies similar to those of gynostemium cells, and gynostemium epidermal cells were smaller compared to WT (Fig. 1B), and in GV2 and GV3, it is the opposite, consistent with the phenotype of severely weakened gynostemium (Fig. S1A). To identify the genetic determinants regulating gynostemium development variation, we reanalyzed previously published GWAS data of 195 *C. sinense* accessions. The analysis revealed multiple loci across the genome that are significantly associated with the GV phenotypes. Among these candidate genes, several MADS-box transcription factors related to flower development were identified (Table S1). However, our previous studies showed that *CsSEP4* is highly correlated with the development of gynostemium in *C. sinense*. Its expression profile in floral organs is also highly associated with the GV phenotypes (Fig. S1A). Subsequently, SNPs with ACGTG/ACGTA/ATGTG variations were detected in the promoter region of the *CsSEP4* gene (Fig. 1C). Specifically, the unique ACGTG type was present in the WT *C. sinense* with normal flower morphology, while the ATGTG and ACGTA types were detected in GV (Fig. S1B). In WT flowers, the *CsSEP4* gene showed the highest expression level in the gynostemium, as compared to other floral organs. However, the overall expression level of *CsSEP4* decreased by more than 70% in GV flowers, and it was evenly distributed in different floral organs, without enrichment in the gynostemium (Fig. 1D and Fig. S1A). Consistent with the majority of MADS-box transcription factors, subcellular localization results showed that CsSEP4 exhibited nuclear localization (Fig. 1E). These results indicated that variation in cis-regulatory elements of the *CsSEP4* promoter was closely related to GV varieties.

### *CsSEP4* regulates the development of gynostemium in *C. sinense*

**Figure 2:**
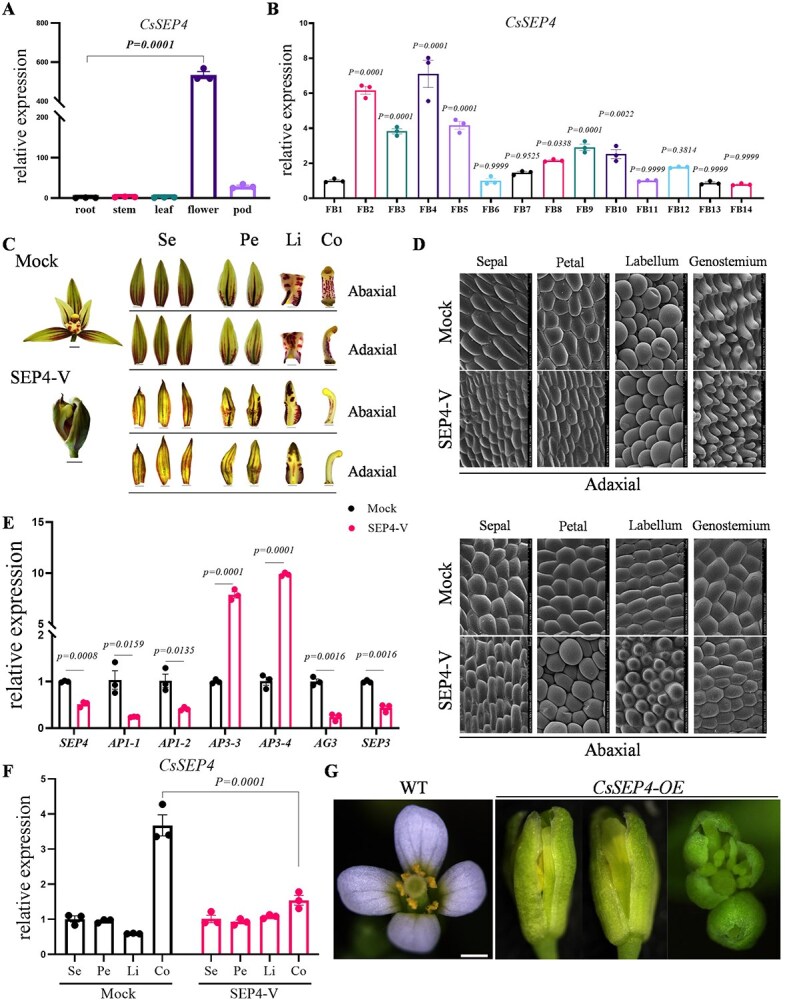
Phenotypic characterization of *CsSEP4* in *Cymbidium* and *Arabidopsis*. (A) Expression of *CsSEP4* in various tissue organs of *C. sinense*. (B) Transcription levels of *CsSEP4* across 14 distinct developmental stages of floral buds during floral development in *C. sinense*. (C) Phenotype observation of flower organ structures transformed with Mock (empty TRV2 vector) and SEP4-V (TRV2-*CsSEP4* vector). Se indicates sepal; Pe, petal; Li, labellum; Co, gynostemium (column). Scale bars: 250 μm. (D) Cell shapes of flowers of Mock and *CsSEP4*-silenced lines by SEM. Bars: 100 μm. (E) RT-qPCR examined the transcript levels of *CsSEP4* and other MADS-box genes (*CsAP1–1/2*, *CsAP3–3/4*, *CsAG3*, *CsSEP3*) in *CsSEP4* silenced lines. (F) Expression profiles of *CsSEP4* in floral organs of mock and silenced lines. (G) Phenotypic analysis of transgenic *Arabidopsis* ectopically expressing *CsSEP4* gene. Scale bars: 100 μm. Data represent the mean ± SEM of three biological replicates, significance is evaluated by the one-way analysis of variance, and *P* values are indicated.

Based on the correlation between the expression profile of *CsSEP4* and the phenotype of GV (Fig. 1A), we further analyzed the biological function of *CsSEP4* in *C. sinense*. Through expression profile analysis, *CsSEP4* exhibited extremely high relative expression levels in floral tissues (Fig. 2A). Extensively, we detected *CsSEP4* transcripts across 14 flower bud developmental stages during floral development and observed high transcription levels from FB2 to FB5 stages in the flower organ formation phase (Fig. 2B and Fig. S2A). Notably, its expression levels at these stages were significantly higher compared with those of other *SEP*-like genes, including *CsSEP1*, *CsSEP2*, and *CsSEP3* (Fig. S2B). Thus, *CsSEP4* is believed to play a critical role in the flower organ formation stage. We used a TRV-mediated virus-induced gene silencing system in *Cymbidium* to characterize the functions of *CsSEP4*. Subsequently, positive silenced lines were identified by PCR and obvious morphological changes were observed in *CsSEP4*-silenced plants (Fig. 2C and Fig. S2C). The gynostemium appeared curved, thinner, and unable to maintain normal development. Particularly, the sepals and petals lacked determinacy and failed to develop normally in *CsSEP4*-silenced lines; they curled inward and were smaller in size. The lip in *CsSEP4*-silenced lines exhibited an outward-extending and flat morphology. Epidermal cells on the adaxial and abaxial surfaces of the gynostemium in silenced plants were much smaller than those in the Mock group, and the cell numbers were higher. Similarly, sepal and petal epidermis cells in the silenced lines had denser distribution, and the cell morphology in the petals became rounder, as compared to those in the Mock group (Fig. 2D). Consistent with these observations, the expression levels of *CsSEP4* were significantly reduced by 50% in *CsSEP4*-silenced lines (Fig. 2E), especially in the gynostemium (Fig. 2F), whereas transcription levels of other *SEP*-like genes (*CsSEP1*, *CsSEP2*, and *CsSEP3*) exhibited no significant changes (Fig. S2D). Floral homeotic genes including the A-class (*CsAP1-1* and *CsAP1-2*), C-class (*CsAG3*), and E-class (*CsSEP3*) genes were significantly decreased, while B-class genes (*CsAP3-3*, and *CsAP3-4*) were significantly upregulated (Fig. 2E). The expression levels of these genes (*CsAP1*, *CsAP3*, *CsAG*, and *CsSEP*) were significantly changed across floral organs, with altered distribution patterns among different whorls in the *CsSEP4*-silenced lines (Fig. S2D and E). This finding further confirmed the regulatory role of *CsSEP4* in gynostemium development in *C. sinense*. Similarly, when *CsSEP4* was ectopically expressed in *Arabidopsis*, phenotypes such as abnormal development of sepals and petals, and carpel-like structures formed from petal transformation were observed (Fig. 2G). Additionally, the qRT-PCR result verified its successful overexpression (Fig. S2F). Combining the above results, we believe that *CsSEP4* plays a significant regulatory function in the morphogenesis of the gynostemium in *C. sinense*. Specifically, its downregulation is associated with developmental abnormalities in the gynostemium, while its upregulation correlates with the formation of ectopic reproductive organs.

### CsbZIP26, a positive regulator, binds to the *CsSEP4* promoter

**Figure 3:**
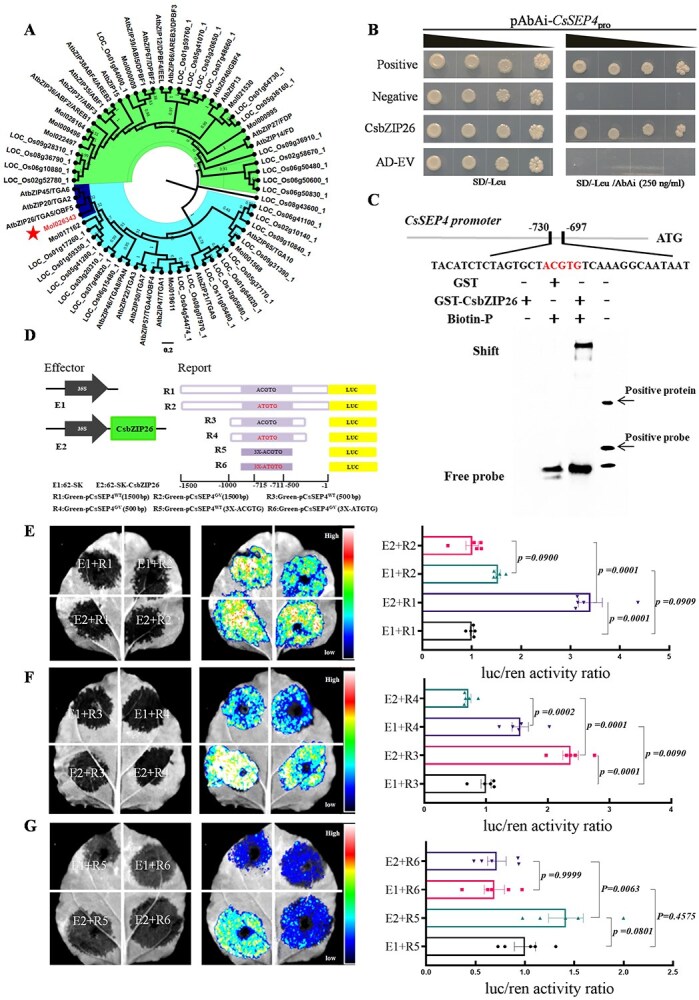
CsbZIP26 binding sites within the *CsSEP4* promoter between WT and GV varieties lead to differences in transcriptional activation efficiency. (A) Phylogenetic analysis was carried out using the amino acid sequences of *CsbZIP26* from *C. sinense* and other homologous proteins. A phylogenetic tree was constructed using the maximum likelihood (ML) method in MEGA7 software with 1000 bootstrap tests. (B) Physical interactions between CsbZIP26 and the promoter of *CsSEP4* through the Y1H system. (C) The binding interaction between CsbZIP26 and the *CsSEP4* promoter using EMSA. The purified GST protein (negative control) or recombinant GST-CsbZIP26 protein with the probe and the formed DNA–protein complexes were separated on a native polyacrylamide gel. (D) Representative effector and reporter constructs were used for the Dual-LUC assay. p*CsSEP4*^WT^ and p*CsSEP4*^GV^ were used as the reporter (R1/2/3/4/5/6). E1 indicates empty vector; E2, SK-CsbZIP26 effector. (E–G) The observed fluorescence signal used luciferase reporter assay via *Agrobacterium*-mediated transformation in *N. benthamiana* leaves. Quantification of the luciferase/*Renilla* (LUC/REN) ratio reflects the LUC activity driven by effector-targeted promoters in *Cymbidium* protoplasts. Data represent the mean ± SEM (*n* = 5 different biological replicates), significance is evaluated by the one-way analysis of variance, and *P* values are indicated.

The SNPs within the promoter of the *CsSEP4* gene, located at −711 to −715, were identified through the GWAS. These SNPs involve mutations from ACGTG in WT to ATGTG or ACGTA in GV (Fig. 1C). To further elucidate how the SNPs in WT and GV flowers regulate the expression of *CsSEP4* in *C. sinense*, Y1H screening was conducted to identify upstream binding proteins of *CsSEP4*. The finding showed that a bZIP family TF, CsbZIP/TGA-like (Mol026343), could bind to the *CsSEP4* promoter. Amino acid sequence alignment and phylogenetic analyses showed that the *CsbZIP/TGA-like* gene in *C. sinense* is closely related to the *AtbZIP26/TGA5/OBF5* gene in *A. thaliana* (Fig. 3A and Fig. S3), which belongs to a well-recognized specific branch related to flower development in other plant species [23]. Consequently, we renamed this gene as *CsbZIP26*. The yeast strains cotransformed with pGADT7-CsbZIP26 and pAbAi-pro*CsSEP4* could grow on the medium supplemented with 250 ng/ml AbAi, while those cotransformed with pGADT7 empty vector and pAbAi-pro*CsSEP4* could not grow on this medium (Fig. 3B). Thus, our results demonstrate that CsbZIP26 possessed the binding ability to the promoter of *CsSEP4* gene. To analyze whether the CsbZIP26 protein can directly bind to the ACGTG motif in target genes *in vitro*, we performed an electrophoretic mobility shift assay (EMSA). The results showed that the purified GST-CsbZIP26 fusion protein could directly target biotin-labeled DNA probes derived from the *CsSEP4* promoter containing the ACGTG element, resulting in the formation of mobility shift bands (Fig. 3C). Subsequently, the dual-luciferase reporter (DLR) assay was employed to compare transcriptional activity between the *CsSEP4* promoter (harboring ACGTG-motif) from WT plants and the *CsSEP4* promoter (containing ATGTG-motif) derived from mutants with severe gynostemium morphological defects (Fig. 3D). Using the promoter fragments of p*CsSEP4*^WT^ (1500 bp) and p*CsSEP4*^GV^ (1500 bp) to drive LUC transcription, we observed that the luciferase signals in *Nicotiana benthamiana* leaves transfected with the *35S:CsbZIP26* effector construct and reporters driven by the promoters of p*CsSEP4*^WT^ (1500 bp) were stronger than those in plants harboring the empty vector. We also performed coexpression assays in *C. sinense* protoplasts and quantified the luciferase/*Renilla* ratio, which further confirmed a significant enhancement of promoter activity. Conversely, reporters driven by the promoters of p*CsSEP4*^GV^ (1500 bp) were weaker compared with the p*CsSEP4*^WT^ (1500 bp) (Fig. 3E), accompanied by a significant suppression of promoter activity. We also used the smaller promoter fragments p*CsSEP4*^WT^ (500 bp), p*CsSEP4*^GV^ (500 bp), p*CsSEP4*^WT^ (3X-ACGTG), and p*CsSEP4*^GV^ (3X-ATGTG) to drive LUC transcription, indicating that the expression of *CsSEP4* can be positively activated by CsbZIP26 in WT normal flowers but can be inhibited in GV mutant flowers (Fig. 3C, F, and  G). Therefore, our results indicated that natural variations in the CsbZIP26 binding sites in the *CsSEP4* promoter between WT and GVs lead to differences in transcriptional activation efficiencies.

### Phenotypic observation of transgenic *Arabidopsis* plants overexpressing *CsbZIP26*

**Figure 4:**
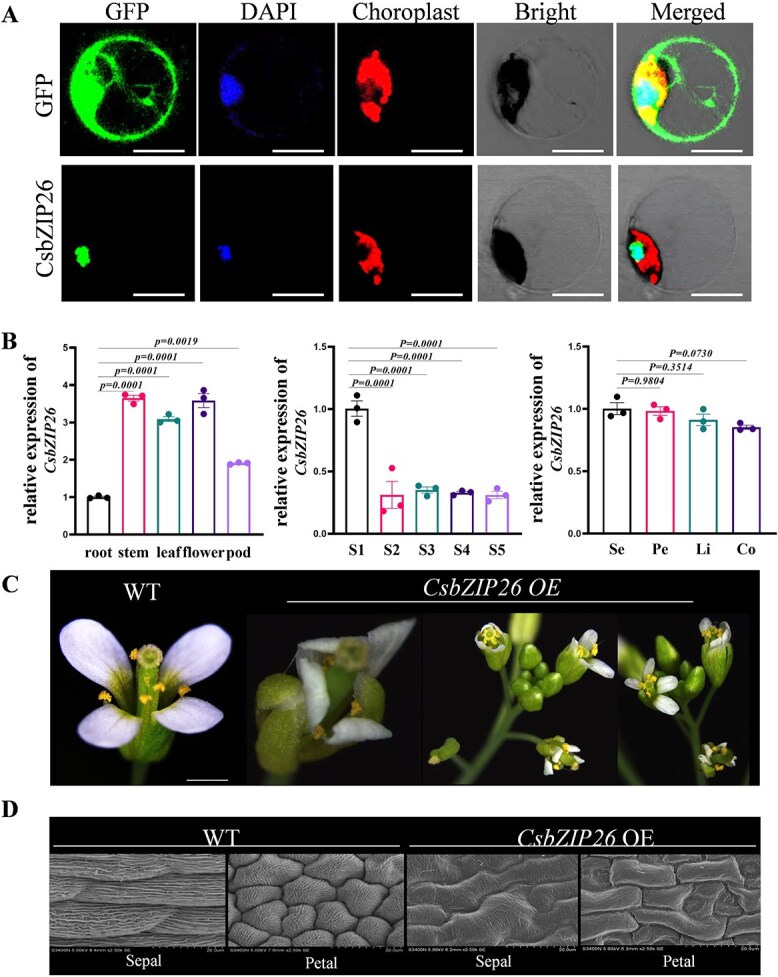
Functional characterization of *CsbZIP26* in *Arabidopsis*. (A) Subcellular locations of *CsbZIP26* in *C. sinense* protoplasts. Scale bars: 20 μm. (B) RT-qPCR analyzed the expression pattern of *CsbZIP26* in *C. sinense*. Data represent the mean ± SEM of three biological replicates, significance is evaluated by the one-way analysis of variance, and *P* values are indicated. (C) Phenotypic analysis of transgenic *Arabidopsis* ectopically expressing *CsbZIP26* gene. Scale bars: 100 μm. (D) Cell shapes of flowers of WT and *CsbZIP26*-OE lines by SEM, Bars: 20 μm.

Based on the differential effects of CsbZIP26 on the transcriptional levels of *CsSEP4* in different varieties, we hypothesize that *CsbZIP26* is crucial for flower development. CsbZIP26 was found to exhibit exclusive nuclear localization through subcellular localization analysis (Fig. 4A), which was consistent with its putative role in transcriptional regulation. The expression profile of *CsbZIP26* demonstrated that it was widely expressed in flower, pod, stem, and leaf organs. Notably, it exhibited the highest expression at flower developmental stage S1 (initiation of floral primordium formation). In mature flowers, the mRNA expression of *CsbZIP26* was uniformly distributed (Fig. 4B). These results indicate that *CsbZIP26* might be involved in the regulation of floral development in *C. sinense*. To elucidate the role of *CsbZIP26* in floral organ regulation, we generated stable transgenic *Arabidopsis* lines overexpressing *CsbZIP26* (Fig. 4C). Subsequent qRT-PCR analysis confirmed the successful overexpression of *CsbZIP26* in these lines (Fig. S3B). Phenotypic analysis demonstrated that overexpression of *CsbZIP26* in *Arabidopsis* affected floral organogenesis. Particularly, the initiation of carpels induced sepal and petal transformation into carpel-like organs. Scanning electron microscopy (SEM) further revealed significant alterations in the epidermal cell morphology of the perianth in *CsbZIP26* transgenic lines. The epidermal cells of sepals and petals exhibited a transformation into carpel-like morphologies (Fig. 4D). These phenotypes were consistent with the ectopic expression of *CsSEP4* gene in *Arabidopsis*.

### Identification of CsSEP4-regulated genes through integrated DAP-seq and transcriptome analysis

**Figure 5:**
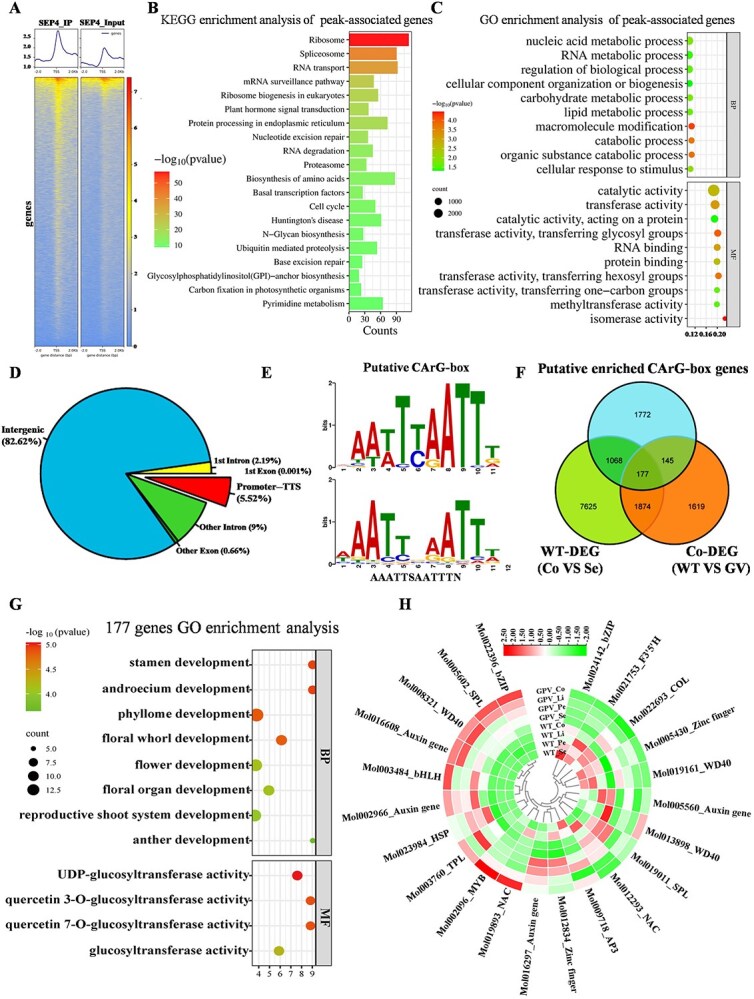
DNA affinity purification sequencing and transcriptome analyses. (A) 2.5k distribution hotspot map upstream and downstream of TSS. (B) KEGG and (C) GO enrichment map of peak related genes. (D) Statistics of distribution regions of binding sites for CsSEP4. (E) Significantly enriched motif sequence of CsSEP4 binding sites specifically detected by DAP-seq. (F) Comparison of DAP-seq and transcriptome data to find high-confidence downstream target genes of *CsSEP4*. The Venn diagram shows that a total of 177 genes were identified. (G) GO enrichment analysis of 177 overlapping genes in (F). (H) The heat map of transcriptional expression of flower development-related genes identified in 177 overlapping genes.

To elucidate the regulatory mechanism of *CsSEP4*, we identified its target genes of CsSEP4 in *C. sinense* through DAP-seq. The results demonstrated that the average unique mapped read ratio was 67.61%, and the average mapped read ratio was 92.30% (Table S1B). CsSEP4 binding peaks showed distinct distribution patterns across different chromosomes (Fig. S4A). Subsequently, we found that the majority of high-confidence binding peaks clustered within 1000 bp of transcription start sites (TSSs) (Fig. 5A and Fig. S4B). Enrichment analyses (KEGG/GO) showed the binding peaks were significantly associated with plant hormone signal transduction, cell cycle, and cellular component organization or biogenesis (Fig. 5B and C). Of the identified peaks, 5.52% were located within a range of 2 kb from the promoter of the annotated genes in the *C. sinense* genome (Fig. 5D). Intriguingly, we found that promoters containing the specific motif (AAATTSAATTTN) are preferentially bound by CsSEP4, which is highly similar to the CArG-box (Fig. 5E). We combined the genes annotated by the peaks to screen out putative target genes located in the promoter regions (Table S2). The result of RNA-seq indicated that these candidate genes are potentially involved in gynostemium morphogenesis (Fig. S4C). Additionally, we performed integrative analysis of the identified CsSEP4-bound genes (DAP-seq) and differentially expressed genes (RNA-seq) between WT and GV varieties to obtain the high-confidence CsSEP4-target genes. By overlapping the differentially expressed genes (DEGs) with CsSEP4 binding genes, 177 potential direct target genes of CsSEP4 were uncovered (Fig. 5F). GO enrichment analysis revealed significant enrichment of these 177 genes in floral organ developmental pathways (Fig. 5G and Table S3). Among them, floral development-associated transcription factors (including SPLs, AP3, bZIP, TPL, HSP, bHLH, WD, MYB, and NAC) showed both highly enriched and high-confidence CsSEP4 binding sites. We found that the expression patterns of *CsHSP*, *CsSPL*, and *CsWD40* genes were correlated with significant upregulation of *CsSEP4* in gynostemium of WT and its downregulation in the GV gynostemium (Fig. 5H).

**Figure 6:**
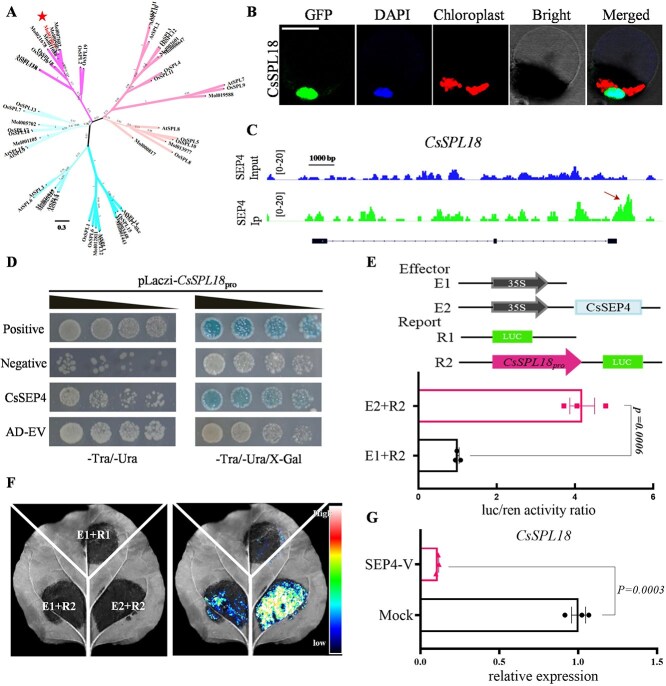
CsSEP4 directly binds to and enhances the transcriptional activity of the *CsSPL18* promoter. (A) Phylogenetic analysis was carried out using the amino acid sequences of CsSPL18 from *C. sinense* and other homologous proteins. A phylogenetic tree was constructed using the maximum likelihood (ML) method in MEGA7 software with 1000 bootstrap tests. (B) Subcellular locations of CsSPL18 in protoplasts of *C. sinense*. Scale bars: 20 μm. (C) CsSEP4-binding profile in the promoter of *CsSPL18*. The arrow represents the distribution of DNA fragment containing the AAATTSAATTTN (motif). DAP-seq reads (IP and Input) were visualized using the IGV program. (D) Y1H assay showing the interactions between the CsSEP4 and the *CsSPL18* promoter. pB42AD empty vector and pB42AD-CsSEP4 were cotransfected separately into the EGY48 cell with pLacZi-*CsSPL18*pro and then grown on the SD/−Trp/-Ura and SD/−Trp/-Ura/X-gal medium. (E) Represented effector and reporter constructs were used for the Dual-LUC assay. *CsSPL18*pro was used as the reporter (R2). The empty SK vector (E1) and the empty reporter vector (R1) served as negative controls, SK-CsSEP4 served as effectors represent (E2). Quantitation of the luciferase/*Renilla* (LUC/REN) ratio, representing the LUC activity of effector targeting the promoter of *CsSPL18* in *Cymbidium* protoplasts. (F) Observation of the fluorescence signal used luciferase reporter assay via *Agrobacterium*-mediated transformation in *N. benthamiana* leaves. (G) The expression level of *CsSPL18* in *CsSEP4* silenced lines. Data represent the mean ± SEM of three biological replicates, significance is evaluated by the *t* test, and *P* values are indicated.

In previous studies, *SPL* genes have been shown to be essential for anther development and morphogenesis of floral organs [29, 39]. To further clarify the characteristics of CsSPL-like protein, we performed BLAST to analyze CsSPL-like and other SBP domain-containing proteins (Fig. S5). Subsequently, the evolutionary divergence of CsSPL-like in the SPL protein family was analyzed and generated a phylogenetic tree (Fig. 6A). Amino acid sequence alignment and phylogenetic analysis revealed that the *CsSPL-like* gene in *C. sinense* is closely related to the *OsSPL18* gene. Therefore, we renamed this gene *CsSPL18*. Subcellular localization assays revealed that the CsSPL18-GFP fusion protein exhibited exclusive nuclear localization (Fig. 6B). Our DAP-seq analysis has identified CsSEP4 can bind to the promoter regions of *CsSPL18* (Fig. 6C). To further elucidate the interaction between CsSEP4 protein and the DNA sequence, we conducted Y1H assays and demonstrated that CsSEP4 could directly bind to the promoter region, which contains the CArG-box binding site (as predicted by the DAP-seq analysis) of *CsSPL18* (Fig. 6D). Subsequently, DLR assays in both *Cymbidium* protoplasts (Fig. 6E) and *tobacco* leaves (Fig. 6F) confirmed that CsSEP4 significantly enhanced the transcriptional activity of *CsSPL18*. In line with these results, the transcript level of *CsSPL18* was dramatically downregulated in *CsSEP4*-TRV silenced lines (Fig. 6G). Consequently, we deduce that the CsSEP4 upregulates *CsSPL18* expression through binding to the CArG-box in its promoter.

### Functional characterization of *CsSPL18* in *Cymbidium* and *Arabidopsis*

**Figure 7:**
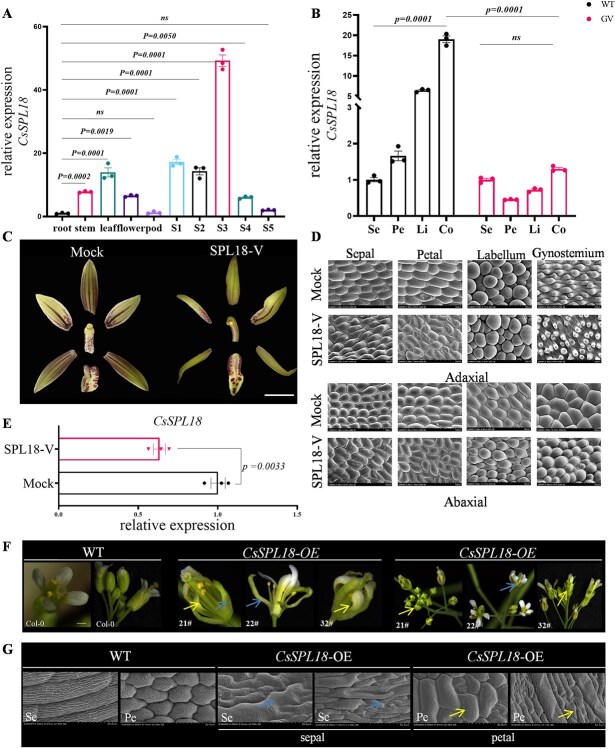
Phenotypic characterization of *CsSPL18* in *Cymbidium* and *Arabidopsis*. (A and B) RT-qPCR analyzed the expression pattern of *CsSPL18* in WT and GV. Significance is evaluated by the one-way analysis of variance, and *P* values are indicated. (C) Phenotype observation of flower organ structure transformed with Mock (empty TRV2 vector) and SPL18-V (TRV2-*CsSPL18*) vectors. Bars: 10 mm. (D) Cell shapes of Mock flowers (empty TRV2 vector) and SPL18-V (TRV2-*CsSPL18* vector) lines by SEM. Bars: 100 μm. (E) RT-qPCR analysis of *CsSPL18* transcript levels in *Cymbidium* VIGS-silenced lines. Significance is evaluated by the *t* test, and *P* values are indicated. (F) Phenotypic analysis of transgenic *Arabidopsis* ectopically expressing *CsSPL18* gene. Arrows indicate carpel-like structures present in both abnormal sepals and petals. Scale bars: 2 mm. (G) Cell shapes of flowers of WT and *CsSPL18*-OE lines by SEM. Arrows indicate the transformation of epidermal cells in sepals and petals. Bars: 20 μm. Data represent the mean ± SEM of three biological replicates.

To gain additional evidence to support the function of *CsSPL18*, we used qRT-PCR to analyze its expression profile in *C. sinense*. Expression pattern analysis indicated that *CsSPL18* was widely expressed in stems, leaves, and flowers, with the highest expression levels detected at the S3 stage of flower development (Fig. 7A). The *CsSPL18* expression was highest in the WT gynostemium across *C. sinense* floral organs (Fig. 7B). However, the *CsSPL18* exhibited downregulation in the gynostemium of GV flowers relative to the WT, while retaining high expression levels in other GV floral structures. This pattern was comparable to the expression profile of *CsSEP4* (Fig. 7B). Subsequently, the *CsSPL18* gene was silenced in *Cymbidium* to explore its function (Fig. 7C). TRV-mediated knockdown of *CsSPL18* disrupted the normal development of the gynostemium, manifested as abnormal stigma contraction and gynostemium curvature, which consequently led to structural deformities during gynostemium formation. Concomitantly, this perturbation induced labellum expansion and perianth inward curling. SEM analysis revealed that petal and labellum cells in *CsSPL18*-silenced lines exhibited cylindrical morphologies similar to those of gynostemium cells, while gynostemium epidermal cells were smaller in size, as compared to Mock (Fig. 7D). The positive silenced lines identified by PCR and RT-qPCR confirmed the transcript levels of *CsSPL18* were significantly downregulated in silenced plants (Fig. 7E and Fig. S6A). In transgenic *Arabidopsis* lines overexpressing *CsSPL18*, abnormal development of floral organs was observed in the first and second whorls (Fig. 7F). The sepals curled and enveloped the floral organs, transforming into carpel-like structures (blue arrows). Similarly, the petals in the second whorl curled and transformed into carpel-like morphologies (yellow arrows). The transcript level of *CsSPL18* following its heterologous expression in *Arabidopsis* was detected by qRT-PCR (Fig. S6B). SEM analysis of perianth epidermal cells in *CsSPL18* transgenic *Arabidopsis* lines revealed morphological changes in both sepal and petal, which resembled carpel cell structures (Fig. 7G). Integrating the above-described mechanism of CsSEP4-mediated positive activation of the *CsSPL18* promoter, we assert that CsSEP4 governs the development of the reproductive organ gynostemium in *C. sinense* by positively regulating the transcriptional level of *CsSPL18*.

## Discussion

*Cymbidium* has been a cornerstone of ornamental horticulture for over two millennia [40]. This genus exhibits a set of evolutionarily distinct traits that are unparalleled in other angiosperms, including specialized floral structures (compact pollen masses, fused androgynophore, modified petal) and highly derived pollinator adaptation syndromes [41]. Among its species, *C. sinense* has given rise to GV varieties, exhibiting pronounced abnormalities in gynostemium development and aberrant floral organ morphologies (Fig. 1A). These distinctive floral characteristics provide crucial insights into both functional conservation and diversification of floral developmental regulation, as well as their special pollination and adaptive reproduction strategies [42, 43]. Although the coevolutionary relationship between orchid floral innovations and specialized pollinators is well documented [12], the molecular mechanisms of these characteristics and insights into the coevolution in *C. sinense* remain poorly understood.

In angiosperms, *SEP* genes represent highly conserved floral organ identity genes that regulate flower development [13, 44, 45]. In this study, by combining the above RNA-seq data and expression pattern analysis in WT and GV varieties of *C. sinense*, we found that the *CsSEP4* gene plays a role in gynostemium development (Fig. 1). In *CsSEP4*-silenced lines, the gynostemium appeared curved and thinner, and was unable to maintain normal development, while heterologous expression of *CsSEP4* induced petal-to-carpel transformation in *Arabidopsis* (Fig. 2). Although similar genes have been reported to be involved in orchid floral development, *CsSEP4* gene in *C. sinense* has undergone distinct subfunctionalization in floral morphology determination compared to its counterparts. For example, previous studies have shown that *PeSEP3* gene promotes the transformation of tepals into leaf-like organs in *Phalaenopsis equestris*, while the *PeSEP4* gene did not show flower organ-determining function [13]. In *C. ensifolium*, the formation of a peloric flower shape with a special lip is regulated by *CsSEP2* [14]. Additionally, the development of floral organs involves a series of cell activities [46]. Our SEM results clearly showed that the shapes of perianth and gynostemium epidermal cells changed in silenced lines (Fig. 2D). As a specialized reproductive organ structure unique to Orchidaceae, the formation of the gynostemium has been extensively documented to enhance pollination efficiency [47]. Interestingly, the gynostemium of the *CsSEP4* silenced lines exhibits axial curvature (Fig. 2C), which may potentially influence the efficiency of pollinia [47]. This phenomenon might be highly correlated with the deceptive pollination strategy prevalent across the orchid family.

Floral organ development is governed by intricate molecular mechanisms that involve coordinated regulation of genetic networks. Among these, the most well-known are the ABCDE model genes [6, 48]. In *CsSEP4*-silenced *Cymbidium* plants, along with the decrease of the *CsSEP4* transcripts, the expression level of the *CsSEP3* also decreased (Fig. 2E), which constitutes a significant factor influencing the phenotypic alterations. In contrast to the downward-curling labellum observed in the WT, the labellum in GV exhibits a relaxed and expanded state (Fig. 1A). Similarly, the flower also features a relaxed rather than curled labellum in *CsSEP4* silenced lines (Fig. 2C). Considering the upregulation of B-class genes (*CsAP3*) in the silenced lines (Fig. 2E) and the previously reported interaction between CsSEP4 and CsAP3, we hypothesize that *CsSEP4* and *CsAP3* mutually regulate to control labellum development [40, 45]. Previous studies have demonstrated that the expansion of B-AP3 class and E-class genes, coupled with their subfunctionalization and neofunctionalization, is evolutionarily linked to the morphological innovation of the orchid labellum and gynostemium [12, 13]. In this study, the *CsSEP4*-silenced phenotype, the expansion of the labellum may amplify visual signaling to pollinators (Fig. 2C), in which phenotypic specialization may represent a critical adaptive component of the deceptive pollination strategies and an evolutionary pattern in floral development [47, 49]. However, the specific regulatory mechanisms governing this process in *C. sinense* remain to be fully elucidated.

Plant bZIP transcription factors exhibit a binding preference toward the ACGT core motif, with TACGTA (A-box), GACGTC (C-box), and CACGTG (G-box/ABRE) motifs, and it exhibits stronger binding affinity for the G-box (ACGTG) than for the A-box (ACGTA) [50, 51]. Here, our investigation identified naturally occurring SNPs within the promoter region of the *CsSEP4* gene between WT (ACGTG) and GV mutant varieties (ATGTG/ACGTA) of *C. sinense* (Fig. 1C). The mild phenotype in GV may be attributed to differential binding affinity of upstream bZIP26 to ACGTG-motif (WT) and ACGTA-motif (GV). However, when the bZIP26 binding motif mutates from ACGTG to ATGTG, it elicits substantial alterations in the regulatory effect on the downstream gene *CsSEP4*. This natural variation mediates differential transcriptional regulation, enabling the upstream transcription factor CsbZIP26 to positively activate the expression of *CsSEP4* in WT but significantly repress its transcription in GV mutant varieties (Fig. 3). Our functional validation also demonstrated that CsbZIP26 binds to the *CsSEP4* promoter to positively activate its transcription, thereby maintaining the formation of gynostemium in WT flowers. In GV varieties, a mutation in the CsbZIP26 binding site reduces its activation of *CsSEP4*, which disrupted the transcriptional level required for *CsSEP4* to maintain normal gynostemium structure (Figs 3 and 4). The expression of specific regulatory networks is often mediated by regulatory elements, a process critical for plant environmental responses and adaptive evolution [52]. Previous studies have demonstrated that mutations in coding regions and cis-regulatory regions frequently drive functional divergence of genes, serving as primary drivers of plant evolution [53, 54].

By establishing a systems-level research framework that integrates developmental mechanisms with ultimate evolutionary patterns, we can elucidate the causal relationships underlying the origins of adaptive complex traits in Orchidaceae [55–58]. In *Arabidopsis*, SPOROCYTELESS (SPL)/NOZZLE, as one of the downstream target genes of the ABCDE functional genes, acts earliest during the late stages of anther differentiation [39, 59]. It has been reported that the MADS-box gene *AtSOC1* promotes flowering by regulating the *AtSPL3*, *AtSPL4*, and *AtSPL5* genes through directly binding to their promoters [31, 32]. In this study, we report the genome-wide identification of CsSEP4 target genes in *C. sinense* using DAP-seq and VIGS assays (Figs 5 and 7). Further analysis combining Y1H and dual-luciferase assays revealed that CsSEP4 plays a role in gynostemium determinacy by directly binding to the promoters of the target gene *CsSPL18*, positively activating and regulating *CsSPL18* expression (Fig. 6). Similar gene functions have been found in previous studies, but they display distinct functions compared to their counterparts in other plants. In *Arabidopsis*, *AtSPL8* maintains male fertility and gynoecium development, while *AtSPL7* is essential for anther and pollen fertility [28–30]. Phenotypic analysis of *CsSPL18* gene in *Cymbidium* also demonstrated its effects on floral organ gynostemium morphology (Fig. 7). Consequently, this molecular mechanism in orchids demonstrates key floral organ identity genes such as the CsSEP4, which likely interacts with numerous downstream MADS-box and non-MADS-box genes to sustain floral organ development. The elucidation of such novel regulatory networks provides insights into gene neo-functionalization and species diversification.

**Figure 8:**
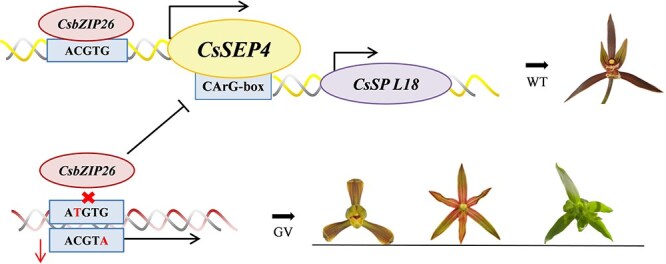
The *CsbZIP26–CsSEP4–CsSPL18* hierarchical module governs morphogenetic innovation in *C. sinense*. In WT plants, CsbZIP26 directly binds to the ACGTG motif in the *CsSEP4* promoter and activates its expression. In GV plants, however, an ATGTG/ACGTA mutation in the *CsSEP4* promoter reduces *CsSEP4* activity, impairing its ability to regulate normal gynostemium development and resulting in the aberrant gynostemium (GV) floral phenotype. Furthermore, CsSEP4 directly binds to the CArG-box motif in the *CsSPL18* promoter to activate its expression, thereby regulating gynostemium development in *C. sinense*.

In this study, we identified a novel regulatory module of *CsbZIP26–CsSEP4–CsSPL18* (Fig. 8), which is crucial for regulating the gynostemium and flower morphology in *C. sinense*. This work provides a more comprehensive and detailed understanding of the functional roles of *SEP* genes in orchids. However, given the complex network of interactions during floral formation in *Arabidopsis*, the development of inner whorls organs (stamens, carpels and ovule) requires the synergistic interaction of B-class, C-class, and E-class MADS-box transcription factors (TFs) [60]. *SEP* genes are indispensable in this process, often serving as scaffolds for higher-order complexes that confer specificity to carpel and ovule development [4]. Notably, this mechanistic paradigm is conserved in diverse plants, such as orchids, where PeSEPs form complexes with class B, C, D, and AGL6-like MADS-box proteins to specify floral organ identity [48, 61]. The assembly of such multiprotein complexes significantly expands the complexity of transcriptional regulatory networks. Beyond MADS-box TFs, gynoecium development involves an extensive regulatory network encompassing over 80 TF families (including *Homeobox*, *ZnF*, *ARF*, *bHLH*, *bZIP*, *MYB*, and *AP2*) that have been implicated in this process, presumably by forming intricate interaction networks [61]. The integrative capacity of SEP proteins is exemplified by *Arabidopsis* SEP3, which directly modulates auxin pathway genes (*ARF*, *AUX/IAA*, *PIN*) and growth-related genes (*TCP*, *GRF*), thereby coordinating floral organ initiation and morphogenesis by linking hormonal signaling and growth regulatory pathways [62]. The ability of the SEP subfamily to form specific protein complexes by interacting with various MADS and non-MADS proteins, modulate target gene expression, and in turn achieve precise tissue-specific transcriptional regulation further suggests that the regulatory network of *SEP* genes in orchid floral development remains highly complex [63]. We speculate that the formation of floral organs in *C. sinense* is not solely determined by the *CsbZIP26–CsSEP4–CsSPL18* pathway. Instead, this process is likely regulated by a more sophisticated network involving a series of floral-related genes. In fact, up to 50% of the target genes may encode proteins with regulatory potential [60, 64, 65]. Nevertheless, the findings of this study will facilitate the elucidation of relationships among developmental genes, establish potential linkages between evolutionary mutations and environmental factors, aid in unraveling the complex mechanisms underlying floral development, and enrich both foundational botanical theories and horticultural applications in orchids.

## Materials and methods

### Plant materials and growth conditions

Normal flowers (WT) and gynostemium variant (GV) of *C. sinense* used in this study were obtained from the greenhouse of the Institute of Environmental Horticulture, Guangdong Academy of Agricultural Sciences. These plants were potted and maintained in the greenhouse. The cultivation conditions for the plants were set at 26°C (day)/23°C (night) under a 16-/8-hour light/dark cycle. *Arabidopsis thaliana* and *N. benthamiana* were cultivated under long-day conditions in an artificial climate chamber for functional analysis.

### Phylogenetic analysis and gene cloning

We retrieved amino acid sequences of genes from a previously published study [40, 60, 64, 65], and other additional genes used for phylogenetic analysis in this work were extracted from National Center for Biotechnology Information. All gene sequences are provided in Table S4. Multiple sequence alignment was carried out using ClustalW default parameters in MEGA v5.2, followed by a maximum likelihood phylogenetic tree that was subsequently constructed [45]. Total RNA was isolated from flower buds of *C. sinense* ‘Baimo’, and then cDNA was synthesized. A list of gene-specific primers is provided in Table S5.

### Quantitative real-time RT-PCR and subcellular localization

RNA was isolated from diverse tissues and organs (roots, stems, leaves, flowers, and pods), flower buds at various developmental stages, and floral organ structures (sepals, petals, labellum, gynostemium) of *C. sinense* ‘Baimo’. Floral bud development was categorized into 5 phases (S1–S5) and further subdivided into 14 more detailed stages (FB1–FB14). The division criteria for the flower bud development stages (S1–S5) referred to those previously published [64]. qRT-PCR analysis was subsequently conducted using the qTOWER 2.0 Real-Time PCR System (Analytik Jena, Germany). The endogenous *β-actin* gene (Mol013347) served as an internal control. All assays were performed with three biological and technical replicates. Subcellular localization was performed as previously described [45]. Primers are detailed in Table S5.

### Plant transformation

The full-length CDS of *CsSEP4*, *CsbZIP26*, and *CsSPL18* were cloned into the pOCA30 to generate overexpression vectors [45]. The plasmids were introduced into competent *Agrobacterium tumefaciens* GV3101 cells (WEIDI, China) by a chemical method, and then the floral dip method was employed to transform *Arabidopsis* [65]. The specific CDSs fragments of *CsSEP4* and *CsSPL18* were inserted into the TRV2 vector. All constructs were transformed into *Agrobacterium*. For transient silencing transformation of *Cymbidium* ‘Golden Rainbow’, the recombinant plasmids were inoculated into flower buds that had not fully completed differentiation (prior to flower bud development stage FB1), at which stage floral organogenesis was incomplete. Following 24-hour incubation at 23°C in the dark, infected plants were moved to a greenhouse for normal growth at 25°C–28°C. At 30 DPI (days post inoculation), the plants showed no nutritional growth retardation, inflorescence determinacy, and no abnormalities in flowering time. Transgenic lines were validated using PCR. Specifically, negative control plants (Mock) were stably transformed transgenic plants harboring the TRV2 empty vector. All experiments were performed at least in triplicate. Primers are detailed in Table S5.

### Yeast-one-hybrid assay

The coding sequence of *CsbZIP26* and the promoter region of *CsSEP4* were constructed into the pGADT7 bait vector and the pAbAi prey vector, respectively, following the methods described earlier [40]. Following cotransformation of recombinant plasmids into the Y1HGold yeast strain, cells were cultured on SD/−Ura medium containing AbA to screen for the optimal concentration of aureobasidin (AbA) required to inhibit the self-activation of the pAbAi recombinant plasmid. Subsequently, the yeast strain was transferred to the SD/−Leu medium supplemented with an appropriate concentration of AbA for cultivation to verify the interaction between the target gene and the promoter. The negative control was the pGADT7 empty vector. Similarly, the coding sequence of CsSEP4 and the promoter region of *CsSPL18* were inserted into pB42AD and pLacZi vectors, respectively. According to the Yeast Protocols Handbook (Clontech), the yeast strain EGY48 was used for cotransformation of recombinant plasmids. Transformants were initially selected on a selection medium (SD/−Trp/−Ura), followed by assessment of transcriptional activity on the plate containing X-gal. All experiments were performed at least in triplicate.

### Dual-luciferase assay

The full-length CDSs of CsbZIP26 and CsSEP4 were inserted into the 62-SK effector vectors, and the promoter regions of *CsSEP4* and *CsSPL18* were separately inserted into pGreenII 0800-LUC vectors. Protoplast transfection was carried out as previously described [45]. Subsequently, the activities of luciferase (LUC) and *Renilla* luciferase (REN) were assessed via a Yeasen luciferase reporter assay kit (Shanghai, China). The *A. tumefaciens* strain GV3101, along with pSuper1300, was transformed with fusion vectors. Following infiltration of *N. benthamiana* leaves with the bacterial suspension, the LUC fluorescent signal was detected after treatment with 1 mM D-luciferin potassium salt.

### Electrophoretic mobility shift assay

The coding sequence of CsbZIP26 and the glutathione S-transferase (GST) tag were inserted in-frame into the pGEX-4 T-1 vector. The GST-CsbZIP26 fusion protein expressed in *Escherichia coli* strain BM Rosetta (DE3) was purified using glutathione–Sepharose 4B beads. The fragment harboring the ACGTG motif within the CsSEP4 promoter was chemically synthesized (Sangon Biotech, Shanghai, China) and biotin labeled at its 5′ end. The EMSA was performed using the LightShift Chemiluminescent EMSA Kit (Thermo Scientific), as described in a previously published study [66]. Following incubation of the biotin-labeled probe with the GST-CsbZIP26 recombinant protein, the bound probe and free probe were separated on a nondenaturing polyacrylamide gel. The GST proteins acted as the negative control.

### DAP-seq analysis

Seqhealth Technology Co., LTD (Wuhan, China), performed the DAP experiment, high-throughput sequencing, and data analysis. The CDS of CsSEP4 was cloned into the pET-28a expression vector using the Cloning Kit (Vazyme, China). Following the manufacturer’s protocol (Promega L3260, USA), we performed protein expression and purification. Genomic DNA from *C. sinense* was extracted, and sheared DNA fragments were then joined with Illumina adaptors using a Vazyme DNA Library Prep Kit for Illumina V3. The DNA fragments were then PCR amplified, purified, quantified, and finally sequenced on Novaseq 6000 sequencer (Illumina) with the PE150 model. Protein binding site analysis was conducted using clean reads. Bowtie2 (v2.2.6) with default settings was used to map reads to the *C. sinense* reference genome. The MEME-ChIP online software (https://meme-suite.org/meme/) was used for peak identification. Homer (version 4.10) was used for motif analysis, followed by integrated transcriptomic [40] analysis to identify downstream candidate genes. The Integrative Genomics Viewer (IGV, https://igv.org/) was used to visualize CsSEP4 binding profiles generated from the DAP-seq assays.

### Scanning electron microscopy

Fully mature flowers were fixed in 4°C glutaraldehyde solution overnight, followed by dehydration in a graded ethanol series and critical point drying. The specimens were coated with gold–palladium and imaged using a Hitachi S-3400N scanning electron microscope at an accelerating voltage of 2 kV.

### Statistical analysis

We used GraphPad Prism 9.10 for statistical analyses, with data reported as mean ± SEM. For two-group comparisons between control and experimental groups, two-tailed Student’s *t* tests were applied. We performed one-way ANOVA with Tukey’s *post hoc* test for comparisons across three or more groups. All experiments were performed at least in triplicate.

## Supplementary Material

Web_Material_uhaf329

## Data Availability

Publicly accessible datasets were subjected to analysis in this research. All original data generated during the study are included in the main text and Supplementary Materials. The raw RNA-seq data generated in this study have been deposited in the National Center for Biotechnology Information (NCBI) Sequence Read Archive (SRA) under the accession number PRJNA743748.
