# Supplementary material for: A novel CsbZIP26–CsSEP4–CsSPL18 regulatory module governs gynostemium morphology and floral architecture in Cymbidium sinense
Source: Hortic Res. 2025 Dec 8;13(3):uhaf329. doi: 10.1093/hr/uhaf329 (PMC12966015; doi:10.1093/hr/uhaf329)
Supplement: Web_Material_uhaf329 [file web_material_uhaf329.zip › 2025.10.24_revision_Supplementary Fig S.pdf]

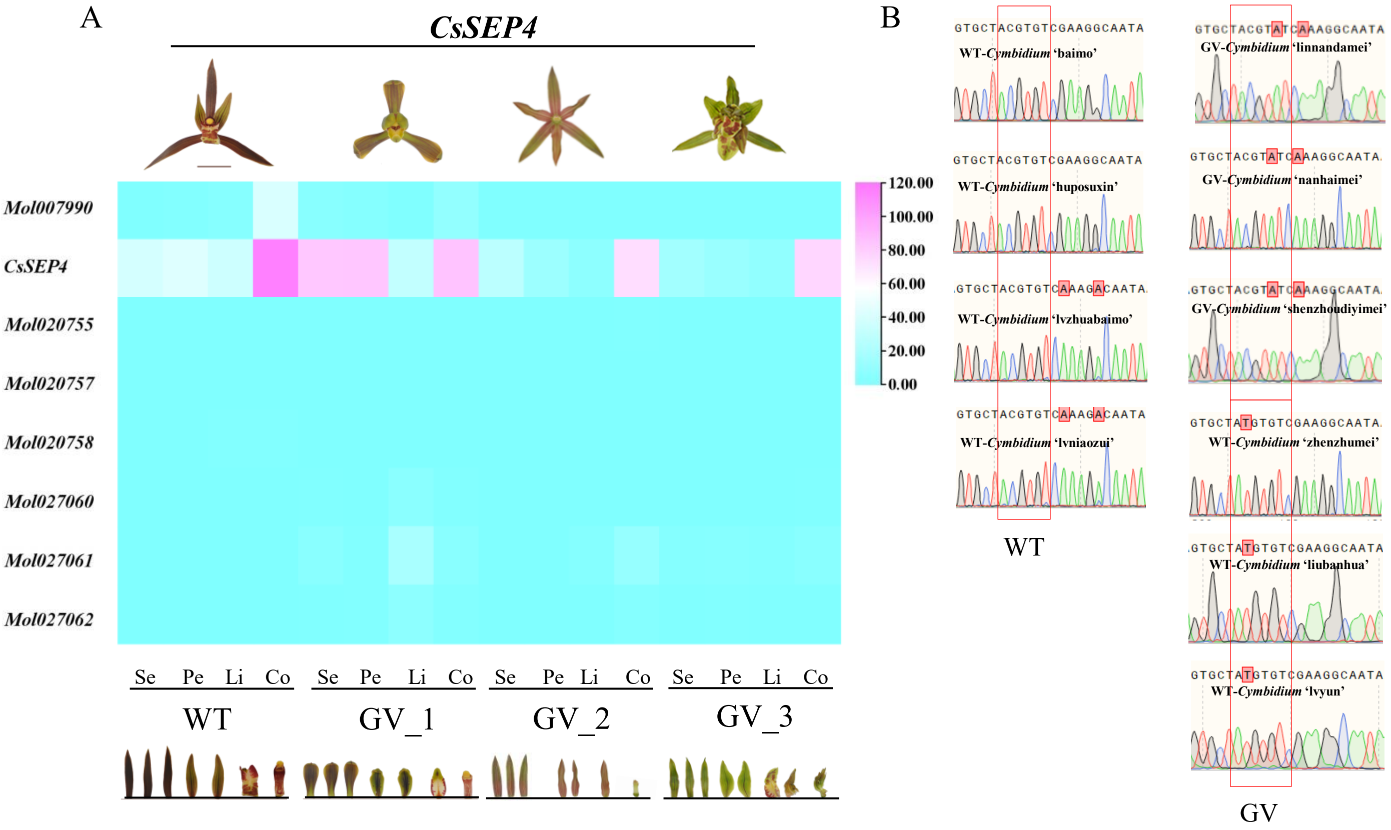

Supplementary Figure S1. (A) The transcript levels of candidate MADS-box genes identified via GWAS were analyzed in flower organ (Sepal, Petal, Lip and gynostemium) of WT and GV varieties. Se: Sepal; Pe: Petal; Li: labellum; Co: gynostemium (Column). (B) The SNPs on the *CsSEP4* promoter in WT and Gynostemium Variant.

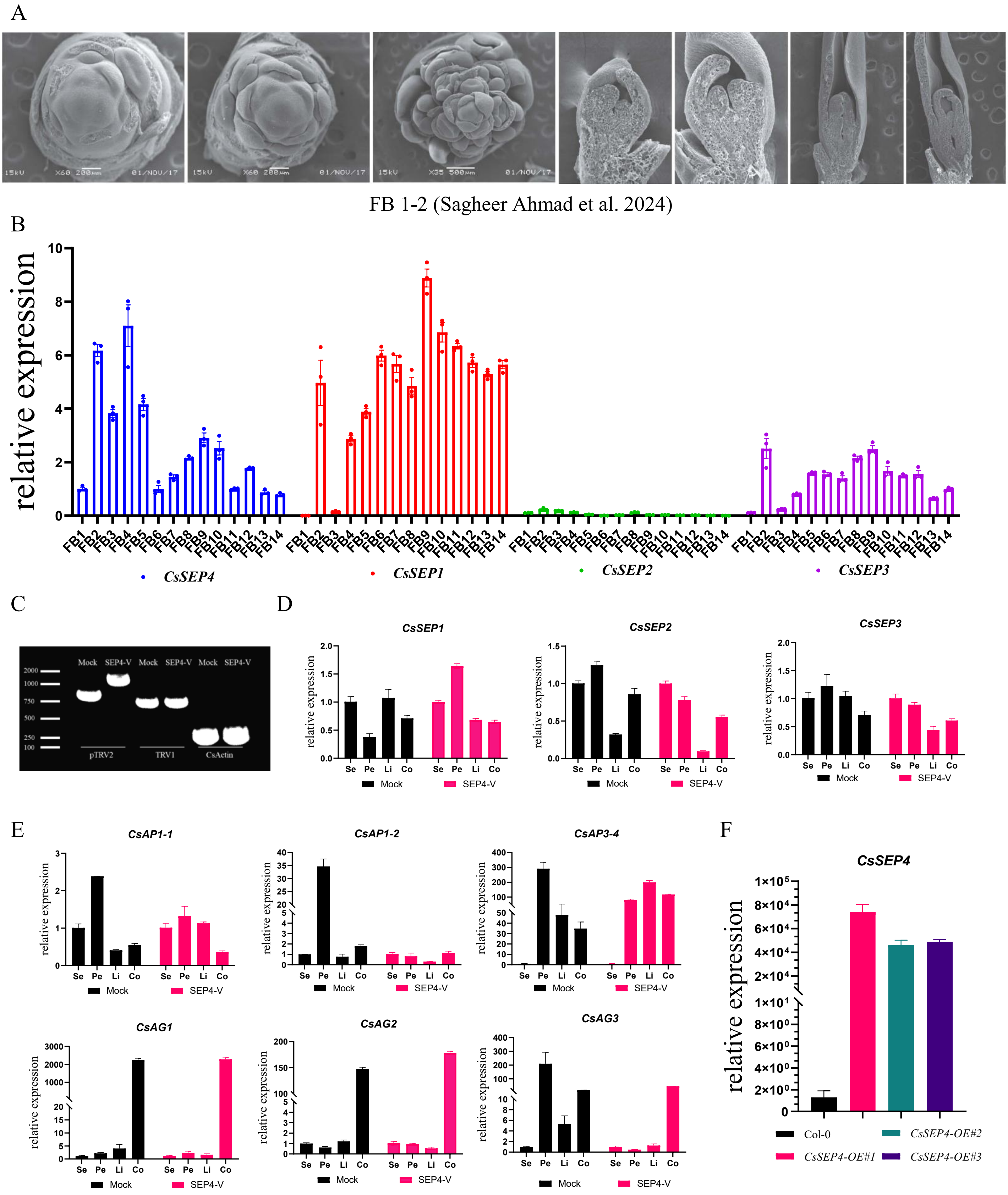

Supplementary Figure S2. (A) The different development stages of flower in *C. sinense*. (B) Expression levels of the *CsSEPs* genes across 14 floral developmental stages of *C. sinense*. (C) PCR identification of plants transformed with Mock (empty TRV2 vector) and SEP4-V (TRV2-*CsSEP4*). TRV1, specific primers in TRV1 vector; TRV2, primers positioned before and after the multiple cloning site of TRV2 vector. (D) The expression profiles of *CsSEP1*, *CsSEP2*, and *CsSEP3* in floral organs of silenced lines. (E) The expression profiles of *CsAP1-1*, *CsAP1-2*, *CsAP3-4*, *CsAG1*, *CsAG2*, and *CsAG3* in floral organs of silenced lines. (F) qRT-PCR analysis of wild-type Col-0 and *35S:CsSEP4* transgenic *Arabidopsis* lines. Data represent the mean ± SEM of 3 biological replicates.

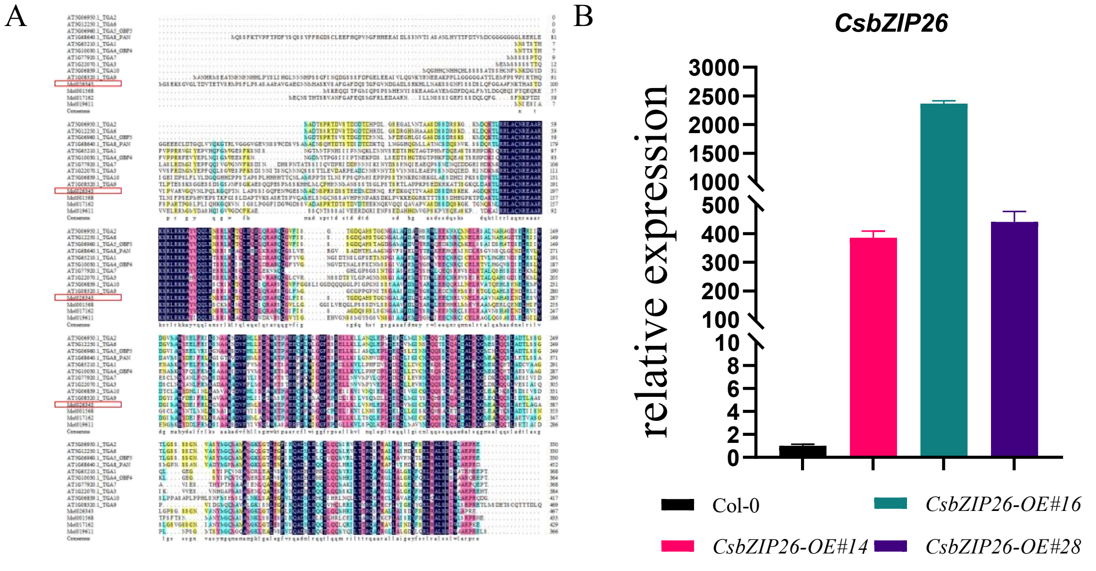

Supplementary Figure S3. (A) Comparative Analysis of Amino Acid Sequences of *bZIPs* genes in *C. sinense* and *Arabidopsis*. (B) qRT-PCR analysis of wild-type Col-0 and *35S:CsbZIP26* transgenic *Arabidopsis* lines. Data represent the mean ± SEM of 3 biological replicates.

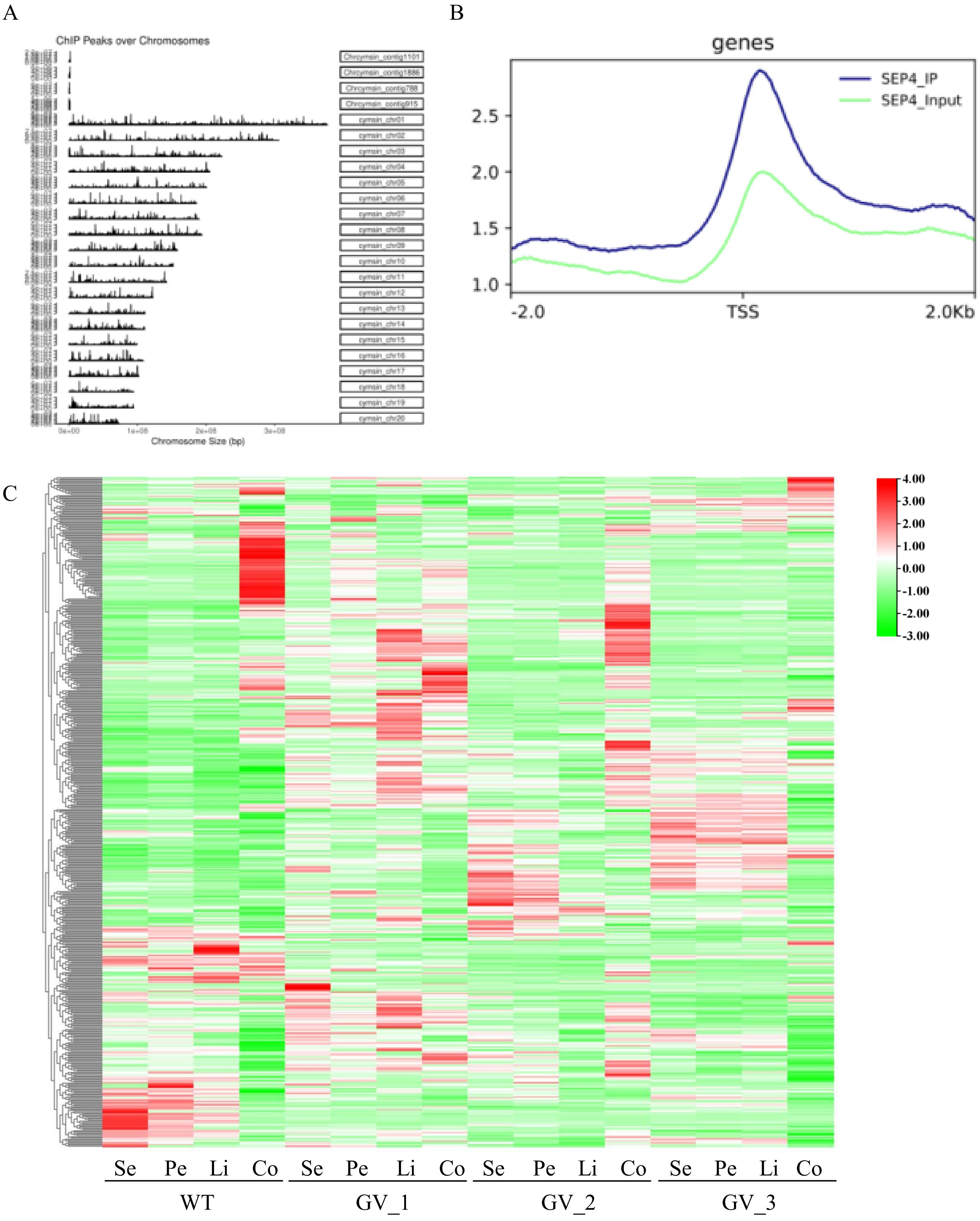

Supplementary Figure S4. DNA affinity purification sequencing analysis.
(A) Distribution of CsSEP4 binding sites on 20 chromosomes of *C.sinense*. (B) Distance from the center of the binding site to TSS for all CsSEP4 target genes. (C) Transcriptome analysis of CsSEP4 target genes with high enrichment peaks identified by DAP-seq.

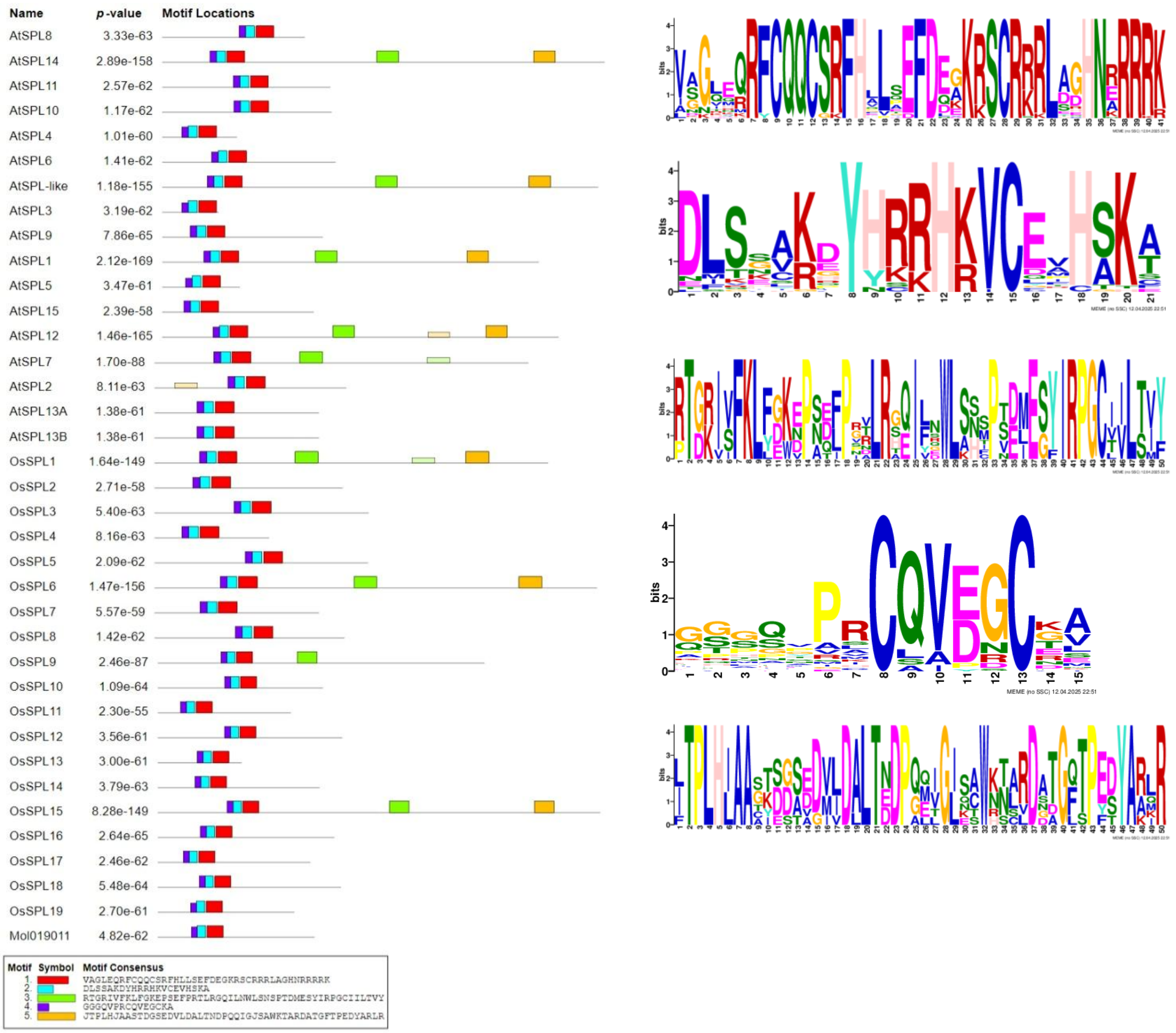

Supplementary Figure S5. BLAST analysis of *CsSPL18* in the nonredundant protein database of NCBI and found other proteins with SBP domain sharing high sequence similarity with *CsSPL18*.

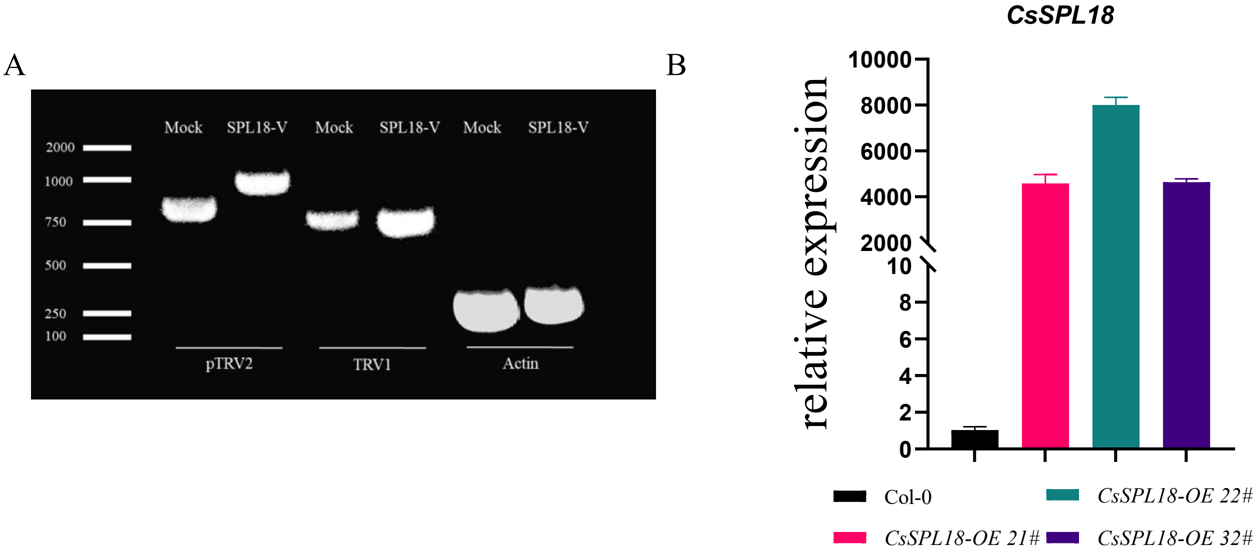

Supplementary Figure S6. (A) PCR identification of plants transformed with empty TRV2 vector (Mock) and TRV2-*CsSPL18* (SPL18-V). (B) RT-qPCR analysis of wild-type Col-0 and 35S:*CsSPL18* transgenic Arabidopsis lines. Data represent the mean ± SEM of 3 biological replicates.
